# Altered responsiveness to TGF-β results in reduced Papss2 expression and alterations in the biomechanical properties of mouse articular cartilage

**DOI:** 10.1186/ar3762

**Published:** 2012-03-06

**Authors:** Girish Ramaswamy, Philip Sohn, Alan Eberhardt, Rosa Serra

**Affiliations:** 1 Department of Biomedical Engineering, University of Alabama at Birmingham, 1530 3rd Avenue South, Birmingham, AL 35294-1150, USA; 2 Department of Cell Biology, University of Alabama at Birmingham, 1530 3rd Avenue South, Birmingham, AL 35294-0005, USA

## Abstract

**Introduction:**

Previous studies have indicated that transforming growth factor β (TGF-β) signaling has a critical role in cartilage homeostasis and repair, yet the mechanisms of TGF-β's chondroprotective effects are not known. Our objective in this study was to identify downstream targets of TGF-β that could act to maintain biochemical and biomechanical properties of cartilage.

**Methods:**

Tibial joints from 20-week-old mice that express a dominant-negative mutation of the TGF-β type II receptor (DNIIR) were graded histologically for osteoarthritic changes and tested by indentation to evaluate their mechanical properties. To identify gene targets of TGF-β, microarray analysis was performed using bovine articular chondrocytes grown in micromass culture that were either treated with TGF-β or left untreated. Phosphoadenosine phosphosynthetase 2 (*PAPSS2*) was identified as a TGF-β-responsive gene. Papss2 expression is crucial for proper sulfation of cartilage matrix, and its deficiency causes skeletal defects in mice and humans that overlap with those seen in mice with mutations in TGF-β-signaling genes. Regulation of Papss2 was verified by real time RT-PCR and Western blot analyses. Alterations in sulfation of glycosaminoglycans were analyzed by critical electrolyte concentration and Alcian blue staining and immunofluorescence for chondroitin-4-sulfate, unsulfated chondroitin and the aggrecan core protein.

**Results:**

DNIIR mutants showed reduced mechanical properties and osteoarthritis-like changes when compared to wild-type control mice. Microarray analysis identified a group of genes encoding matrix-modifying enzymes that were regulated by TGF-β. *Papss2 *was upregulated in bovine articular chondrocytes after treatment with TGF-β and downregulated in cartilage from DNIIR mice. Articular cartilage in DNIIR mice demonstrated reduced Alcian blue staining at critical electrolyte concentrations and reduced chondroitin-4-sulfate staining. Staining for unsulfated chondroitin sulfate was increased, whereas staining for the aggrecan core protein was comparable in DNIIR and wild-type mice.

**Conclusion:**

TGF-β maintains biomechanical properties and regulates expression of Papss2 and sulfation of glycosaminoglycans in mouse articular cartilage.

## Introduction

Osteoarthritis (OA) is the most common form of arthritis and a major cause of disability worldwide. OA is primarily a disease that affects articular cartilage, the permanent cartilage present on surfaces of diarthrodial joints. It is important for smooth functioning and load transfer across the joints. Chondrocytes respond to a variety of stimuli, including mechanical loading and growth factors that maintain cartilage homeostasis. Type II collagen and proteoglycans, primarily aggrecan, are the major constituents of the extracellular matrix (ECM), which form a meshwork that acts as the main load-bearing component of the cartilage [1].

The transforming growth factor β (TGF-β) superfamily is known to play an important role in the skeletal system, especially in the development and maintenance of growth plate and articular cartilage [2,3]. Altered signaling and reduced expression of TGF-β ligands and receptors have been associated with OA in both mice and humans [4,5]. Previously, it was shown that mice expressing a dominant-negative mutation of the TGF-β type II receptor (DNIIR) in the cartilage have OA-like symptoms, including increased hypertrophy, chondrocyte clustering and osteophytes in the joint space [6]. Similar results were previously shown using mouse models with alterations in other components of TGF-β signaling, including Smad3, LTBP3 and Smurf2 [7-9]. None of these studies characterized the changes in biomechanical properties of articular cartilage during joint degeneration, however, and the mechanisms of TGF-β's chondroprotective effects are still not known.

Biomechanical integrity is critical for healthy functioning of the joints. Changes in extracellular and pericellular matrix, water content and fixed-charge density are significant features of OA and are known to affect the mechanical properties of cartilage [10-12]. Cartilage matrix contains high concentrations of negatively charged sulfate and carboxyl groups that help attract and retain water during loading [13]. Sulfation is an essential posttranslational modification in which sulfate groups are added to glycosaminoglycan chains that are covalently linked to core proteins of proteoglycans. 3'-Phosphoadenosine 5'-phosphosulfate synthetase 2 (Papss2) is a bifunctional enzyme that catalyzes the synthesis of 3'-phosphoadenosine-5'-phosphosulfate (PAPS), the universal sulfate donor for all sulfotransferase reactions [14,15]. Mutations in *PAPSS2 *cause an autosomal recessive form of spondyloepimetaphyseal dysplasia (SEMD), Pakistani type [OMIM:612847], in humans, whereas a point mutation in the adenosine 5'-phosphosulfate kinase region of *Papss2 *causes brachymorphism (bm) in mice [16-19]. Both conditions are characterized by short stature, kyphoscoliosis and premature joint degeneration that resemble some of the phenotypic features of mice with altered TGF-β signaling [6,17,19-21]. Factors that regulate *Papss2 *expression are unknown.

The goal in this study was to identify downstream targets of TGF-β that act to maintain biochemical and biomechanical properties of cartilage. We identified *PAPSS2 *as a TGF-β-regulated gene in bovine articular cartilage. We subsequently showed that Papss2 and the level of chondroitin 4-sulfate are downregulated in DNIIR cartilage, whereas levels of the aggrecan core protein are comparable with those of control mice. We hypothesize that altered TGF-β signaling and the resulting changes in Papss2 expression and matrix sulfation adversely affect the mechanical properties of the cartilage, leading to joint degeneration.

## Materials and methods

### DNIIR mice

All mice in this study were maintained and handled with the approval of the Institutional Animal Care and Use Committee of the University of Alabama at Birmingham. Generation of transgenic mice expressing a cytoplasmic truncated, functionally inactive DNIIR under the control of a metallothionein-like promoter was described previously. Mice were genotyped by PCR with DNA extracted from mice tails. The primer sequences for genotyping were described previously [6].

### Mechanical testing

**Figure 1:**
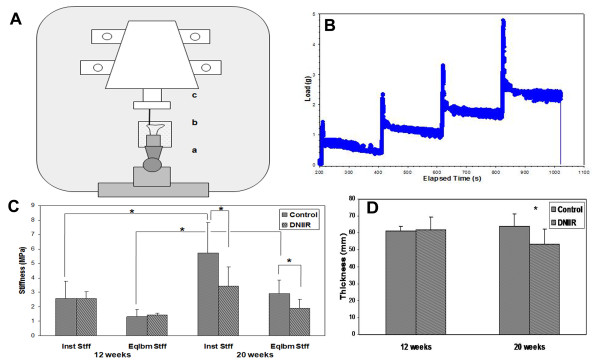
**Mechanical properties of articular cartilage**. **(A) **Schematic of indentation testing setup. **(a) **360° rotating arm. **(b) **Specimen chamber filled with PBS. **(c) **250-g load cell with indenter. **(B) **Representative four-step stress relaxation curve with 5-μm displacement steps and 200-second relaxation time between each step. **(C) **Indentation results for control and dominant-negative transforming growth factor β type II receptor (DNIIR) tibiae at ages 12 weeks (*n *= 3 controls and 3 DNIIR) and 20 weeks (*n *= 7 controls and 6 DNIIR). Significant changes in stiffness were not observed in DNIIR cartilage at 12 weeks. Instantaneous and equilibrium stiffness were significantly lower in DNIIR cartilage at 20 weeks. In control mice, stiffness increased significantly between ages 12 and 20 weeks. Stiffness was not significantly changed for DNIIR mice over the same time period. **(D) **Articular cartilage thickness was significantly reduced in DNIIR mutants as compared to age-matched controls at 20 weeks of age, but no difference was observed at 12 weeks. Values are means ± SD. *P < 0.05.

The mice were killed at 12 or 20 weeks of age, and right tibiae were extracted and stored at 4°C in PBS until tested. Mechanical testing was done within 48 hours of tissue extraction. Articular cartilage was tested by indentation on a computer-controlled electromechanical test system (Bose LM1 ElectroForce TestBench; Bose Corp, Eden Prairie, MN, USA) fitted with a 250-g load cell (Sensotec Honeywell, Columbus, OH, USA). The tibiae were cut with a surgical blade at 6 mm measured from the articular cartilage surface, embedded in polymethylmethacrylate, mounted in a custom-made specimen chamber, immersed in PBS and maintained at room temperature. The articular surface was maintained wet with PBS during the embedding process. The specimen chamber was fixed on a custom-made X-Y stage with micrometer control and a 360° rotating arm (Figure 1A).

A four-step stress relaxation test was performed on each specimen utilizing a custom-made cylindrical, impervious, plane-ended indenter (178-μm diameter) positioned perpendicular to the cartilage surface with the use of a stereomicroscope. Indentation was performed on the lateral tibial plateau. The indenter was positioned approximately at the central region of the tibial surface with respect to the mediolateral and dorsoventral axes. Indentation was performed consistently at the same location by viewing it through a stereomicroscope and adjusting the sample by moving the X-Y stage so that the indenter was on top of the central region of the lateral tibial plateau. Initially, a tare load of 0.05 g was applied and the cartilage was allowed 200 seconds to reach equilibrium. The cartilage surface was then displaced by 20 μm over four steps of 5 μm each with a relaxation time of 200 seconds between every step (Figure 1B). Load values measured immediately after every displacement step and at the end of every 200-second equilibrium period were converted to stress values by dividing the load by the indenter surface area, and strain values were calculated using the ratio of displacement steps to cartilage thickness [10,22]. Stress-strain curves were plotted separately for both instantaneous and equilibrium values. Stiffness was determined as the slope of the respective curves and called instantaneous and equilibrium stiffness, respectively. After stress relaxation was complete, the load was removed and the cartilage was allowed to return to its original shape. The indenter was replaced with a sharp tungsten needle (1-μm tip radius) that was pushed through the cartilage surface to measure cartilage thickness as indicated by changes noted in the load-displacement curve at the surface and tidemark [23].

### Histology

Tibiae from mice were fixed in 4% paraformaldehyde overnight, decalcified, processed in ethanol solutions and embedded in paraffin. Sections 6 μm thick were cut and mounted on Superfrost Plus slides (Fisher Scientific, Pittsburgh, PA, USA). Sections were stained with H & E, toluidine blue and Sirius red as described previously [24]. Sections were stained employing the critical electrolyte concentration principle with 0.05% Alcian blue 8GX (Electron Microscopy Sciences, Hatfield, PA, USA) in 0.025 M sodium acetate buffer at pH 5.8 and 0.5 M MgCl_2 _overnight and were counterstained with nuclear fast red [25,26].

### Bovine micromass cultures

Bovine metacarpal-phalangeal joints were obtained from a local abattoir. Cultures were set up according to a protocol described previously [27,28]. Briefly, full-thickness slices of cartilage were taken off the bone and placed into DMEM/10% fetal bovine serum (FBS) containing antibiotics and fungicide. The tissue was then minced finely and placed into DMEM/10% FBS containing 0.2% collagenase overnight. The isolated cells were removed, washed, strained and resuspended in fresh DMEM/10% FBS at a concentration of 2 × 10^7 ^cells/ml. Twenty-microliter drops were placed into the bottom of each well of a 24-well plate. The cells were allowed to adhere to each other and to the plate for 3 hours, then the well was flooded with 1 ml of DMEM/0.5% FBS, ascorbate and antibiotics.

Alcian blue staining was quantified as the absorbance at 595 nm of the guanidine-extracted stain over the quantity of DNA measured by the fluorescence of Hoechst dye [29]. Cells were washed, stained with 0.125% Alcian blue in 0.1 M HCl overnight at 37°C, washed and extracted in 6 M guanidine hydrochloride. Alkaline phosphatase activity was quantified as nanomolar *p*-nitophenol released per hour over the amount of DNA.

### Affymetrix microarrays

RNA was isolated from bovine cells grown in micromass culture either untreated or treated with 5 ng/ml TGF-β1 (R&D Systems, Minneapolis, MN, USA) for 8 hours using TRIzol reagent (Invitrogen, Carlsbad, CA, USA). The Affymetrix GeneChip Bovine Genome Array (Affymetrix, Inc, Santa Clara, CA, USA) was completed in the Gene Expression Shared Facility located in the Heflin Center for Genomic Sciences at the University of Alabama at Birmingham. The quality of each RNA sample was determined by analysis on the 2100 Agilent Bioanalyzer (Agilent Technologies, Inc, Santa Clara, CA, USA) prior to RNA labeling. Detailed GeneChip analytical procedures are presented in the Manufacturer's GeneChip Expression Technical Manual (Affymetrix). Briefly, 50 ng of total RNA from each sample were used in a two-cycle cDNA amplification protocol using T7-linked oligo(dT) primers according to the manufacturer's instructions. After the first round of cDNA synthesis, an *in vitro *transcription step was utilized to amplify the RNA, following which a second round of cDNA synthesis was performed. Subsequently, cRNA was generated and biotin was incorporated into the cRNA strand using standard methods (Affymetrix), followed by cRNA fragmentation and preparation of hybridization cocktail. The arrays were hybridized overnight at 45°C, then washed, stained and scanned the next day. Gene expression levels were extracted using the Affymetrix GeneChip Command Console (AGCC; Affymetrix).

Statistical analysis and gene lists for the array experiments were generated using the GeneSpring software package (Agilent Technologies). Briefly, to generate gene lists, the raw GeneChip files (.cel) from GeneChip Operating Software (AGCC) were uploaded to GeneSpring, background was subtracted and data were normalized using the Robust Multichip Average method and default settings in GeneSpring. The untreated control group was used as a baseline to calculate the intensity ratio or fold changes of the TGF-β-treated versus control groups. The ratio was log_2_-transformed before further statistical analysis. The *P*-values were calculated by *t*-test assuming unequal variance. Microarray data were deposited into the Gene Expression Omnibus database [GEO:GSE29233].

### Real-time RT-PCR

RNA from bovine cultures was used for real-time RT-PCR to confirm the array results. To verify regulation in DNIIR mice, RNA was isolated from articular cartilage from wild-type (WT) and DNIIR mice. First, articular cartilage was carefully dissected from forelimb and hind limb joints and digested in 3 mg/ml collagenase D for 3 hours at 37°C, and cells were isolated from the matrix. RNA was extracted from the cells using TRIzol reagent (Invitrogen). RNA was DNase-treated (Promega, Madison, WI, USA), and real-time RT-PCR was performed using the SYBR Green RT-PCR Kit (QIAGEN, Valencia, CA, USA) on the LightCycler 480 Real-Time PCR System (Roche Applied Science, Indianapolis, IN, USA). Changes in expression levels of bovine or mouse *Prg4, Papss2*, and *Plod2 *were determined using hypoxanthine ribosyltransferase (*Hprt*) as the normalization gene in mouse samples and glyceraldehyde 3-phosphate dehydrogenase (*GAPDH*) in bovine samples. Primers that crossed intron boundaries were either designed or selected from PrimerBank http://pga.mgh.harvard.edu/primerbank/. The sequences are listed in Table 1. The results were analyzed using REST software [30].

**Table 1:** Primer sequences used in real-time RT-PCR

Gene	Forward primer	Reverse primer
Mouse		
*Hprt*	TCA GTC AAC GGG GGA CAT AAA	GGG GCT GTA CTG CTT AAC CAG
*Prg4*	GAA AAT ACT TCC CGT CTG CTT GT	ACT CCA TGT AGT GCT GAC AGT TA
*Papss2*	CCC GTG ATG GAG TCA TCA ACA TGA G	GTG CTT TGC AGT GGC TGT TCC
*Plod2*	CAC TGG ATT ATC CCA AAG AAG CCC	CCT GGC TTC TGC TTG ACT TAG GTT
*Fxyd2*	ATC CCT TCG AGT ACG ACT ATG AA	CAG CGG AAC CTT TTG CTG AGA
Bovine		
*Gapdh*	GGG TCA TCA TCT CTG CAC CT	GGT CAT AAG TCC CTC CAC GA
*Prg4*	TGC CCT GAC TTC AAG AAG GAA TGC	CCA TAA TCG GAA CAG CAC TTG CCA
*Papss2*	TGC CAT CTT CCC ATC TCC CAT GTT	ACA GGT CTC TCT TGG TCT CAG GAT
*Plod2*	CCC TTT CCT ACC TCG ATT TCT GAA CAC	CCA TGT TTC TGG CTT CAG CTT GAC

### Western blot analysis

Articular cartilage from forelimb and hind limb joints of 6 week, 20 week, and 10 month old control and DNIIR mice was digested for 3 hours in 3 mg/ml collagenase D (Roche Applied Science) dissolved in DMEM/F-12 nutrient mixture (Gibco/Life Technologies, Grand Island, NY, USA) at 37°C in a thermal incubator. The digest was filtered through a 40-μm cell strainer. After centrifugation, the pellet was lysed in TRIzol reagent (Invitrogen) and both RNA and protein were extracted according to the manufacturer's instructions. Equal amounts of protein samples from the cell lysate were separated by reducing electrophoresis with 10% acrylamide gels. After transfer to polyvinylidene fluoride membrane (Bio-Rad Laboratories, Hercules, CA, USA), the membranes were blocked with 5% nonfat dry milk and incubated with monoclonal antibody to Papss2 (1:1,000 dilution; Sigma-Aldrich, St Louis, MO, USA). Blots were then incubated with horseradish peroxidase-conjugated goat anti-mouse immunoglobulin G (IgG) (Santa Cruz Biotechnology, Santa Cruz, CA, USA). Immunoblots were developed by chemiluminescence (Thermo Fisher Scientific). Gapdh was used as a loading control. The blots were scanned using an Epson Perfection V700 photo scanner (Epson America Inc, Long Beach, CA, USA) at 1,200 dpi, then densitometry was measured using KODAK molecular imaging software (Eastman Kodak, Rochester, NY, USA).

### Immunofluorescence

Paraffin-embedded tibial sections from 20-week-old mice were dewaxed and hydrated. Antigen unmasking was performed by incubating sections in 0.1% trypsin (Sigma-Aldrich) in 1 × PBS at 37°C for 12 minutes. Sections were treated with chondroitinase ABC (Sigma-Aldrich) for 1 hour at 37°C. The Mouse on Mouse (M.O.M.) Immunodetection Kit (Vector Laboratories, Burlingame, CA, USA) was then used with mouse primary antibodies, and immunofluorescence was performed according to the manufacturer's instructions. Sections were stained with anti-chondroitin-4-sulfate (C4S) (2-B-6) and anti-chondroitin-0-sulfate (C0S) (1-B-5) (Seikagaku Corp, Tokyo, Japan) at 1:400 dilution. Biotinylated anti-mouse IgG secondary antibody, followed by Cy3-conjugated streptavidin, was applied to the sections. Sections without chondroitinase treatment were included for antibody specificity. For aggrecan, after antigen-unmasking sections were blocked with 5% BSA and 5% goat serum in Tris-buffered saline with 0.1% Tween 20 and incubated with anti-rabbit aggrecan antibody (AB1031; Millipore, Billerica, MA, USA), and Alexa Fluor 488-conjugated anti-rabbit secondary antibody was used for fluorescence. Sections without primary antibody were included for antibody specificity.

### Statistical analysis

Indentation test results were assessed by analysis of variance to compare 12- and 20-week control and mutant samples. *P *< 0.05 was considered statistically significant. Real-time RT-PCR data were analyzed using REST software [30], which enables statistical comparison of different genes between multiple groups. Instead of applying a parametric test, REST software performs randomization tests (at least 2,000 randomizations) on groupwise data and calculates expression ratios of target genes compared to a reference gene along with the *P*-values. Differences were considered significant at *P *< 0.05.

## Results

### Mechanical properties of articular cartilage in control and DNIIR mice

Previously, we showed that dominant-negative interference of Tgfbr2 (DNIIR) in mice results in OA-like symptoms in the peripheral joints [6]. To characterize any TGF-β-mediated alterations in the mechanical properties of articular cartilage in mice, a four-step stress-relaxation-indentation test was performed on the tibial plateau of 12- and 20-week-old WT control and DNIIR mice (Figures 1A and 1B). No significant differences in stiffness or cartilage thickness were observed between WT and DNIIR mice at 12 weeks of age (Figures 1C and 1D). In contrast, both instantaneous and equilibrium stiffness were significantly reduced (about 35%, *P *< 0.05) (Figure 1C) in 20-week-old mutant mice compared to age-matched WT controls. In addition, stiffness was significantly increased in control mice between 12 and 20 weeks of age; however, the increase in DNIIR mice between ages 12 and 20 weeks was much less and not significant (Figure 1C). Articular cartilage thickness as measured by the needle probe method showed that mutants also possessed thinner articular cartilage (about 17%, *P *< 0.05) (Figure 1D) relative to controls at 20 weeks of age. The structural stiffness results should not be affected by this small change in cartilage thickness, because stiffness is calculated as the slope of the stress-strain curve, where strain is the change in cartilage thickness divided by the original thickness. The results suggest that alterations in TGF-β signaling adversely affect the mechanical properties of articular cartilage in mice.

**Figure 2:**
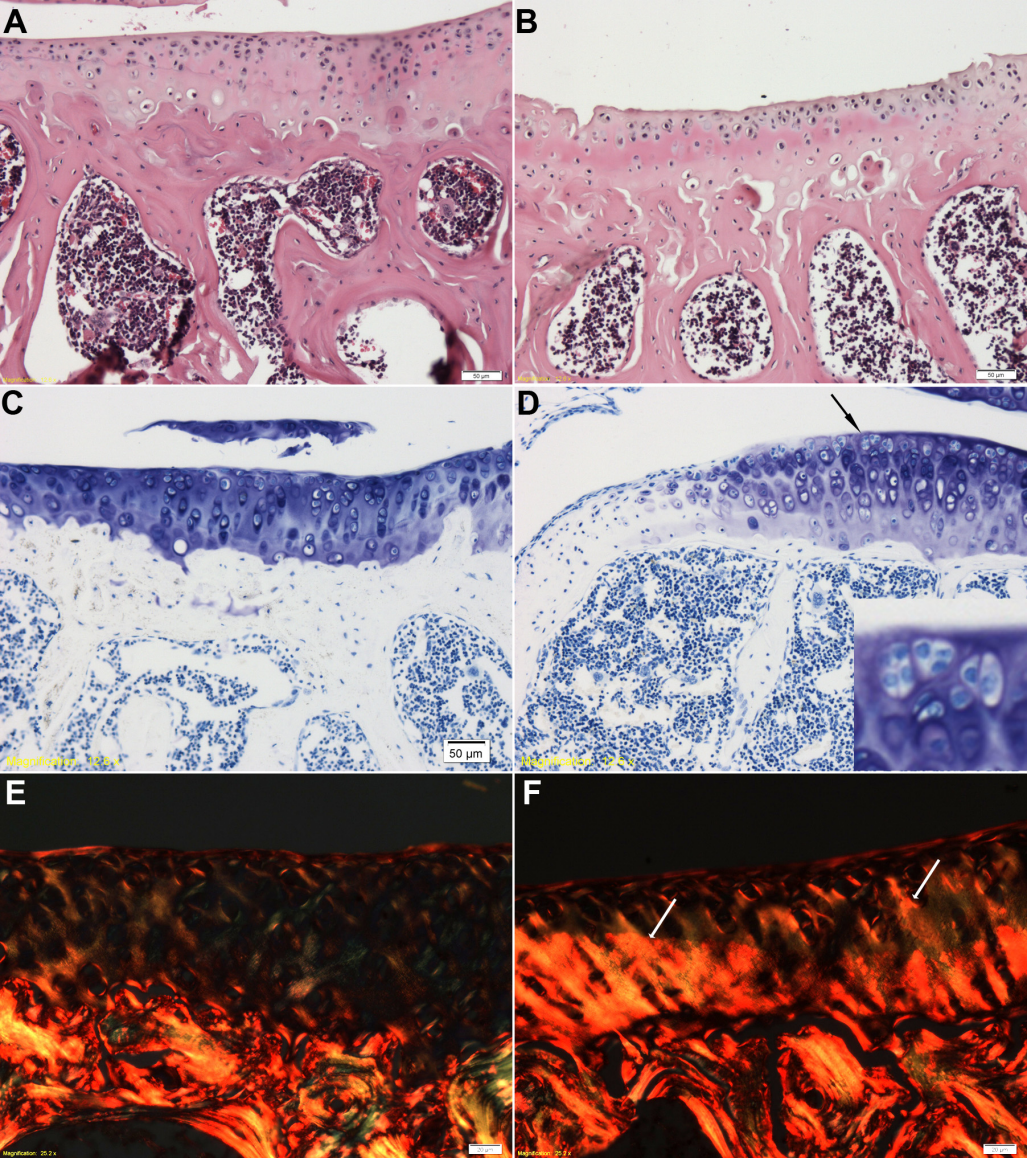
**Mild osteoarthritis observed in dominant-negative transforming growth factor β type II receptor mice**. Sections from 20-week-old control **(A)**, **(C) **and **(E) **and dominant-negative transforming growth factor β type II receptor (DNIIR) **(B)**, **(D) **and **(F) **tibiae (*n *= 3 control and 3 DNIIR) were stained with H & E (A) and (B), toluidine blue (C) and (D) and Sirius red/polarized light microscopy (E) and (F) to determine the extent of osteoarthritis (*n *= 3 controls and 3 DNIIR). Fibrillation at the surface (B), focal cationic matrix stain depletion (D), cell clusters (D, arrow and inset) and disorganized and increased collagen fibers (F, arrows) are shown.

To determine the severity of OA in the mice tested, we took sections of the contralateral tibiae from a subset of 20-week-old mice (*n *= 3 control and 3 DNIIR) and stained them with H & E to determine the basic histology of the tissue, with toluidine blue to determine the level of proteoglycan matrix depletion and with Sirius Red by polarized light microscopy to determine the level of collagen organization (Figure 2). The cartilage exhibited matrix discontinuity at the superficial layer to shallow vertical fissures by H & E staining (Figures 2A and 2B). Loss of toluidine blue staining was focal but went deep into the cartilage (Figures 2C and 2D). Clustering of chondrocytes was evident in the top layers of the cartilage. Collagen fibers were disorganized, and there was an increase in collagen staining suggestive of new collagen formation (Figures 2E and 2F). To determine the extent of OA in the mutant mice, we used the scoring system described by Pritzker *et al. *[31]. In this system, grade describes the depth of OA and stage describes how much of the surface is involved. The final score is calculated as grade time stage. Because more than 50% of the cartilage surface was involved in 20-week-old DNIIR mice, the stage was 4.0. The grades of DNIIR mice at the same age were 2.0 to 3.0, so the scores for 20-week-old DNIIR mice were 8.0 to 12.0. The score for WT mice was 0. The extent of OA in these mice was less severe than previously reported [6], likely due to a selective advantage for breeding in mice with less severe OA.

### Matrix-modifying genes are regulated by TGF-β in articular cartilage

**Figure 3:**
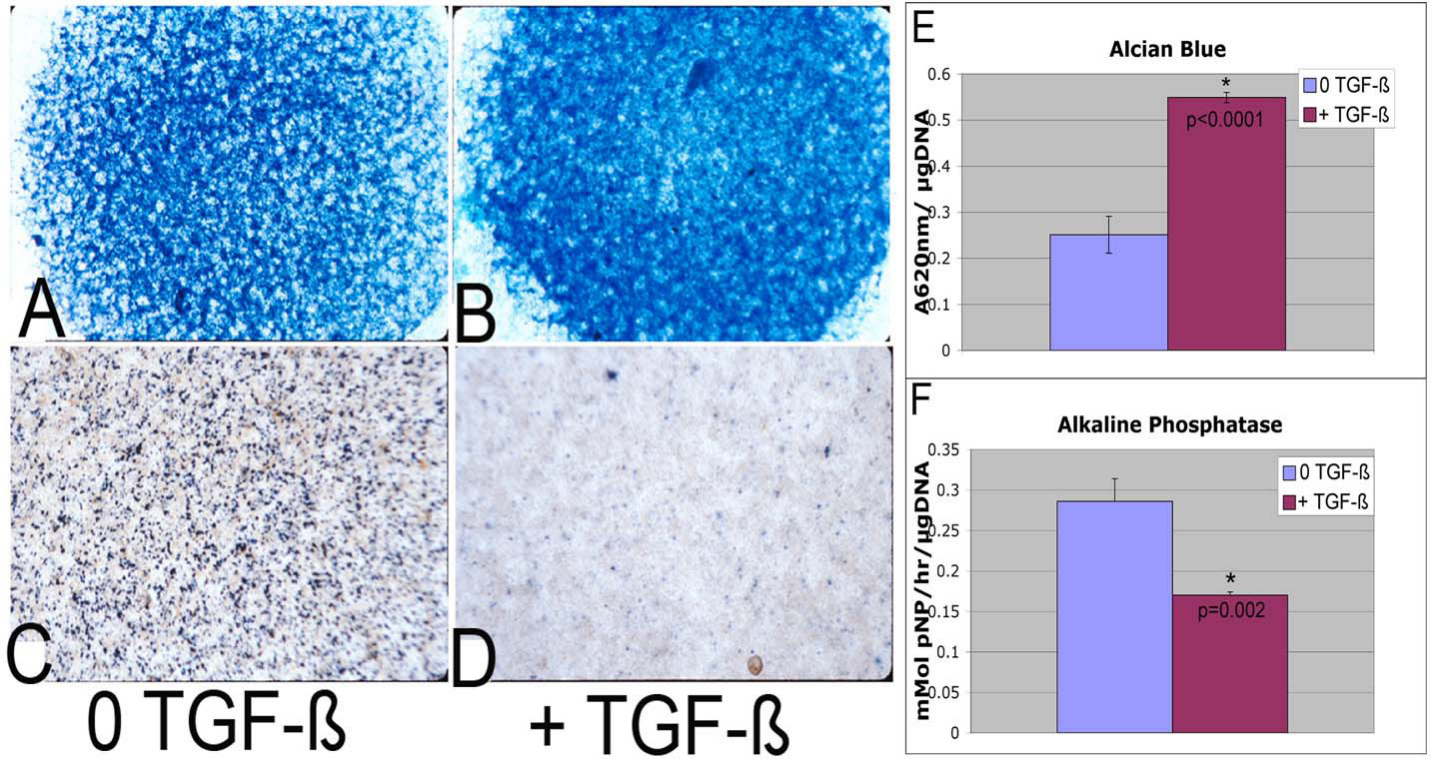
**Bovine articular chondrocytes in micromass culture**. Bovine articular chondrocytes grown in micromass cultures were left untreated **(A) **and **(C) **or treated with transforming growth factor β (TGF-β) **(B) **and **(D) **for 7 days. Cultures were stained for Alcian blue (A) and (B) or alkaline phosphatase (C) and (D). Quantification of staining showed an increase in Alcian blue staining **(E) **and a decrease in alkaline phosphatase activity after treatment with TGF-β **(F)**. *P-value < 0.05 by *t*-test. pNP: *p*-nitophenol.

The data above and previous results suggest that TGF-β normally prevents joint degeneration; however, the downstream effectors of TGF-β in the articular cartilage are not known [6-9]. To identify genes that are likely direct targets of TGF-β in the articular cartilage, we used bovine articular chondrocytes grown in micromass culture [27,28]. This strategy has the advantages over dissecting tissue from mice that there is less contamination with other cell types and the genes identified are more likely to be directly regulated by TGF-β. The effect of TGF-β on the phenotype of the chondrocytes in culture was tested first (Figures 3A through 3D). Cells were treated with 5 ng/ml TGF-β1 for 7 days. Standard Alcian blue staining was used as a measure of overall proteoglycan content (Figures 3A and 3B). The level of staining was then quantified by absorbance at 595 nm (Figure 3E). Alkaline phosphatase staining (Figures 3C, D and 3F) was used as a measure of late hypertrophic differentiation. Treatment with TGF-β resulted in increased Alcian blue staining and decreased alkaline phosphatase activity, indicating that TGF-β is active in this model. This is consistent with data previously reported for bovine articular chondrocytes treated with TGF-β [28].

Next, RNA was isolated from cells untreated or treated with 5 ng/ml TGF-β1 for 8 hours. Microarray analysis was done using Affymetrix GeneChip Bovine Genome Array GeneChips, which contain 24,072 probe sets representing approximately 23,000 bovine genes. Micromass cultures of articular cartilage from three separate cows were grown in the presence or absence of TGF-β, resulting in three biological replicates for each condition. The results were analyzed using GeneSpring software and the default statistical cutoffs. A gene list was generated that contains genes that were regulated either up or down by at least twofold with a *P*-value < 0.05. There were 60 downregulated genes on the list, and 65 genes were upregulated. A list of selected genes is shown in Table 2. A full list of all the regulated genes is given in Additional file 1. The list includes several genes known to be regulated by TGF-β, including proteoglycan 4/lubricin (*PRG4*) [32], parathyroid hormone-like hormone (*PTHLH*) [33], TGF-β-induced protein IG-H3 (*TGFBI*) [34] and prostate transmembrane protein, androgen-induced 1 (*PMEPA1*) [35], suggesting that the microarray was effective in identifying TGF-β-regulated genes. Surprisingly few genes encoding structural components of the ECM were identified; however, several matrix-modifying genes were upregulated, including 3'-phosphoadenosine 5'-phosphosulfate synthase 2 (*PAPSS2*), procollagen lysine 2-oxoglutarate 5-dioxygenase 2 (*PLOD2*), Β-1,3-*N*-acetylgalactosaminyltransferase 2 (*B3GALNT2*), UDP-gal:βGlcNAcβ1,3-galactosyltransferase polypeptide 2 (*B3GALT2*), glycosyltransferase 8 domain-containing 2 (*GLT8D2*), dermatan sulfate epimerase (*DSE*) and arylsulfatase family, member I (*ARSI*). We also recently identified *Papss2 *as regulated by TGF-β in a microarray screen of TGF-β-treated mouse embryonic sclerotome [36]. This result suggested that TGF-β may function to maintain articular cartilage strength by regulating posttranslational modifications in ECM proteins.

**Table 2:** Selected genes regulated by TGF-β in bovine articular chondrocytes

Probe set ID	Gene name	Gene symbol	Fold difference	Direction
TGF-β-regulated				
4670.1.S1_at	Transforming growth factor-β-induced protein IG-H3	*TGFBI*	10.44	Up
668.1.S1_at	Prostate transmembrane protein, androgen-induced 1	*PMEPA1*	3.5	Up
22987.1.A1_at	Proteoglycan 4/lubricin	*PRG4*	2.11	Up
12848.1.S1_at	Parathyroid hormone-like hormone	*PTHLH*	4.12	Up
Matrix				
568.1.S1_at	Integrin-binding sialoprotein	*IBSP*	4.48	Down
25236.1.A1_at	Matrix-remodeling-associated 5	*MXRA5*	3.35	Up
Protein-modifying				
28724.1.A1_a_at	Arylsulfatase family, member I	*ARSI*	3.88	Up
7447.1.A1_at	UDP-gal:βGlcNAc β-1,3-galactosyltransferase, polypeptide 2	*B3GALT2*	2.43	Up
5086.2.S1_a_at	β-1,3-*N*-acetylgalactosaminyltransferase 2	*B3GALNT2*	2.2	Up
17755.1.A1_at	Dermatan sulfate epimerase	*DSE*	2.53	Up
24824.1.S1_at	Glycosyltransferase 8 domain-containing 2	*GLT8D2*	3.6	Down
1087.1.S1_at	3'-phosphoadenosine 5'-phosphosulfate synthase 2	*PAPSS2*	2.67	Up
25109.1.S1_at	Procollagen lysine 2-oxoglutarate 5-dioxygenase 2	*PLOD2*	2.1	Up

**Figure 4:**
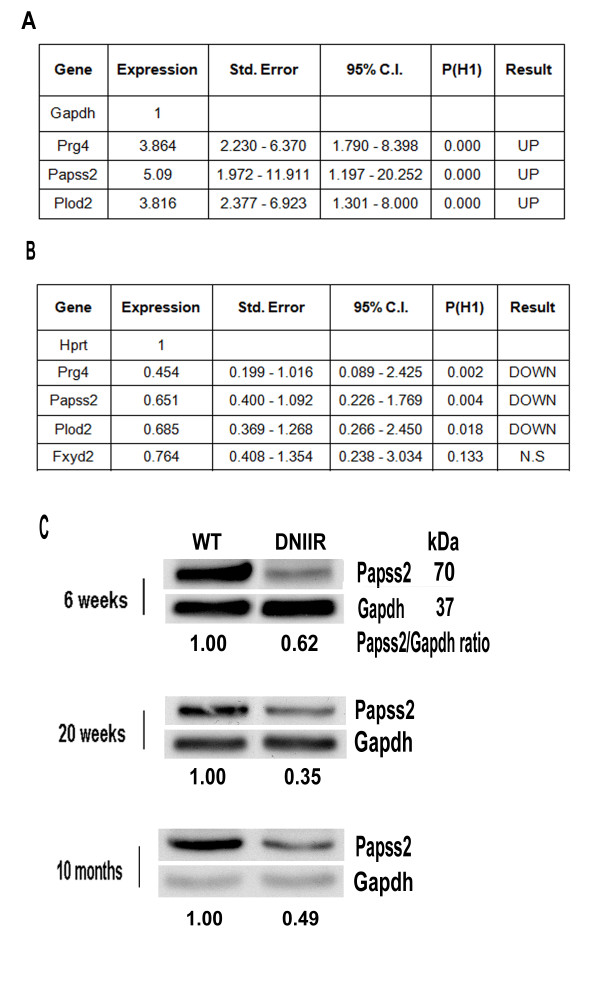
**Papss2 is regulated by transforming growth factor β**. **(A) **RNA isolated from bovine chondrocytes grown in micromass cultures and either left untreated or treated with 5 ng/transforming growth factor β (TGF-β)/ml for 8 hours (*n *= 3 separate cows) was used in real-time RT-PCR. Data are shown as tables obtained using REST software [30]. *Papss2 *and two other selected genes (*Prg4 *and *Plod2*) were upregulated after treatment with TGF-β, confirming the microarray results. Gapdh was used as a normalization control. **(B) **Gene expression was compared by real-time RT-PCR using RNA isolated from 20-week-old wild-type (WT) and dominant-negative TGF-β type II receptor (DNIIR) mice (*n *= 5 controls and 6 mutants) using REST software. Significant downregulation of *Prg4, Papss2 *and *Plod2 *was seen in DNIIR samples. *Hprt *was used as a normalization control. *Fxyd2 *was not regulated by TGF-β on the microarray, which we verified by RT-PCR. **(C) **Cell protein lysates were isolated from articular cartilage of 6-week-old, 20-week-old and 10-month-old control and DNIIR mice (*n *= 3 each at age 6 weeks, 4 each at age at 20 weeks and 2 each at age 10 months). Papss2 expression was determined by Western blot analysis. Gapdh was used as a loading control. The average Papss2/Gapdh ratios derived from all of the blots are shown, with the ratio for WT set to 1. Papss2 protein was reduced in DNIIR mice at each stage.

We verified regulation of several genes by TGF-β in bovine articular cartilage micromass cultures using quantitative real-time RT-PCR (Figure 4A). Gene expression was determined in cultures generated from three separate cows, with each sample generated in triplicate. Combined PCR data from the three biological replicates were analyzed using REST software, which normalizes results and calculates the relative fold differences in gene expression, confidence intervals and statistical significance across experiments using an integrated randomization and bootstrapping algorithm [30]. Bovine micromass cultures treated with TGF-β showed significant upregulation of *PRG4*, a known target of TGF-β (about 3.9-fold), *PAPSS2 *(about fivefold) and *PLOD2 *(about 3.8-fold) relative to untreated controls, thus verifying the microarray results.

### Papss2 expression and sulfation of chondroitin are reduced in DNIIR cartilage

Next, to determine if reduced TGF-β signaling in DNIIR mice would alter expression of these genes *in vivo*, quantitative real-time RT-PCR was performed as described above with RNA from the articular cartilage of 20-week-old DNIIR and WT mice (*n *= 5 WT and 6 DNIIR). Expression of DNIIR mRNA was confirmed in each sample by semiquantitative RT-PCR (data not shown). Downregulation of *Prg4 *(mean mRNA expression ratio of DNIIR/WT = 0.454), a known TGF-β-responsive gene, was used to confirmed disruption of TGF-β signaling in DNIIR cartilage (Figure 4B). Significant downregulation of *Papss2 *(DNIIR/WT = 0.651) and *Plod2 *(DNIIR/WT = 0.685) was observed in DNIIR cartilage as compared to WT (Figure 4B). *Fxyd2 *encodes an ion transport regulator, which was used as an unregulated control. The results suggest that TGF-β signaling is required to maintain expression of these genes *in vivo*.

Skeletal alterations in mice and humans with deficiencies in Papss2 have similarities to those of mice with deficiencies in TGF-β signaling, including kyphosis, postnatal short stature and progressive degeneration of articular cartilage. Therefore, we decided to focus on Papss2 [6,16-21,37]. To check whether reductions in Papss2 mRNA translated to changes in protein levels, we performed Western blot analysis using protein lysates from cells isolated from articular cartilage of 6-week-old, 20-week-old and 10-month-old mice. When Papss2 levels in DNIIR mice were compared to those in controls, the mutants exhibited close to a 50% reduction in Papss2 protein levels at all ages tested (Figure 4C). These results suggest that TGF-β is required to maintain protein levels of Papss2 in cartilage.

**Figure 5:**
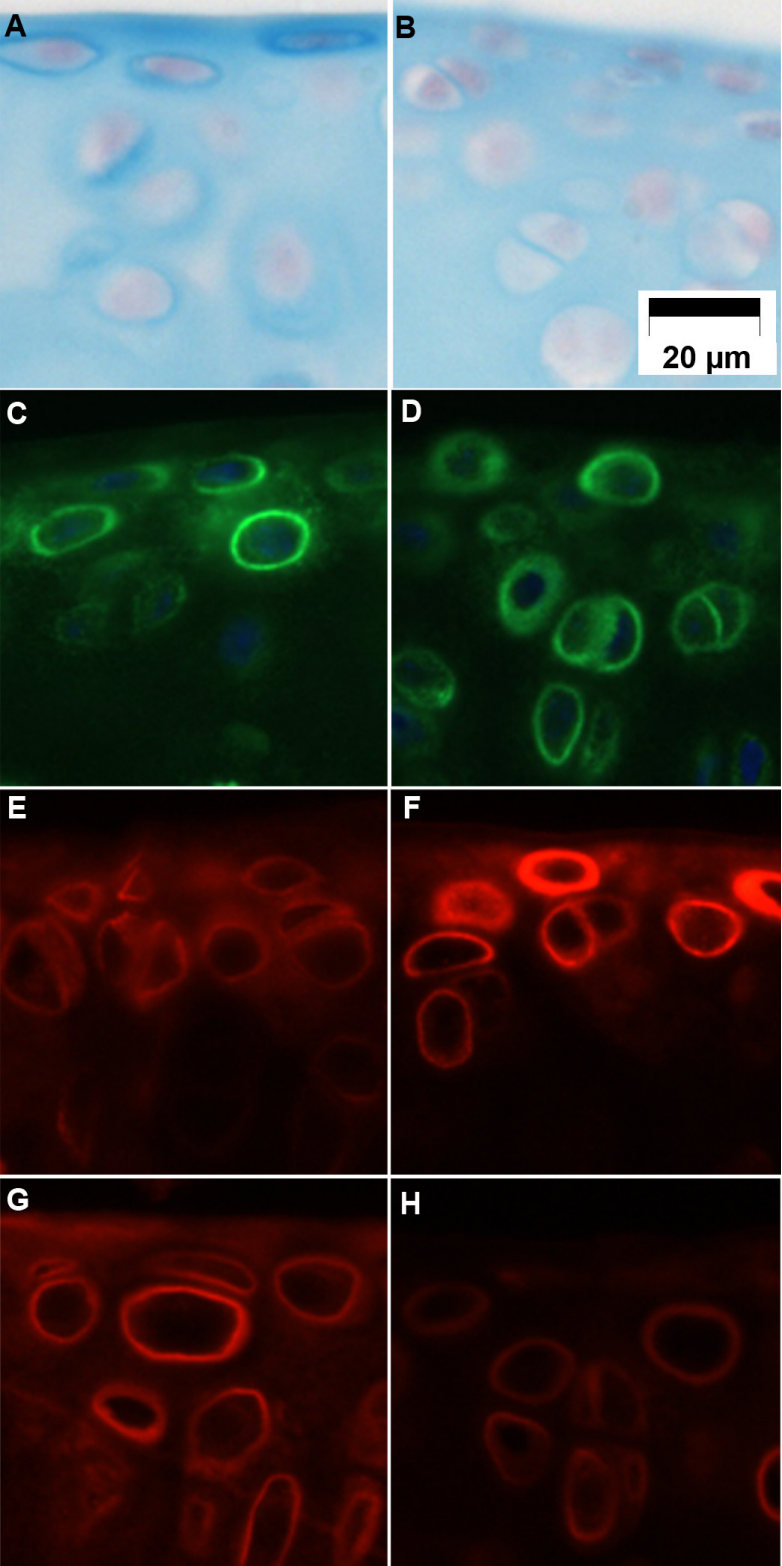
**Chondroitin-4-sulfate is reduced in DNIIR cartilage**. Sections from wild-type **(A)**, **(C)**, **(E) **and **(G) **and dominant-negative transforming growth factor β type II receptor (DNIIR) **(B)**, **(D)**, **(F) **and **(H) **tibiae (*n *= 3 controls and 3 DNIIR) were stained using Alcian blue at critical electrolyte concentration (A) and (B) and immunofluorescence using anti-aggrecan (C) and (D) (green), anti-chondroitin-0-sulfate (E) and (F) (red) and anti-chondroitin-4-sulfate (G) and (H) (red) antibodies. Alcian blue critical electrolyte staining and chondroitin-4-sulfate staining were reduced in DNIIR cartilage, chondroitin-0-sulfate staining was increased and aggrecan staining was comparable to that of controls.

Alcian blue at specific molar concentrations of MgCl_2 _(0.5 M) and pH 5.8 has been shown to specifically stain sulfated glycosaminoglycans with preference for chondroitin sulfate. This is known as critical electrolyte concentration (CEC) Alcian blue staining [25,26]. To investigate whether DNIIR cartilage had alterations in sulfation of glycosaminoglycans, we first compared CEC Alcian blue staining in sections from 20-week-old WT and DNIIR tibiae (*n *= 3 WT and 3 DNIIR) (Figures 5A and 5B). In sections from WT mice, deep blue staining could be seen in the pericellular matrix surrounding cells as well as in the superficial layers of the cartilage. Some blue staining was also observed in the interterritorial matrix of the deeper zones. In contrast, deep blue staining was reduced or absent in the pericellular matrix in DNIIR cartilage. In addition, staining appeared to be reduced in the superficial zone.

To determine if the reduced staining was simply due to lower levels of aggrecan core protein, we stained separate sections from the same samples by immunofluorescence using an anti-aggrecan core protein antibody (Figures 5C and 5D). In both controls and mutants, staining could be seen at comparable levels in both the pericellular and interterritorial matrix; therefore, the changes in CEC Alcian blue staining could not be accounted for by alterations in aggrecan core protein levels.

Next, we looked directly at C0S (Figures 5E and 5F) and C4S (Figures 5G and 5H) using antibodies that detect terminal unsaturated disaccharide sulfated in the C-4 position of *N*-acetylhexosamine residue attached to carbohydrate stub of the core protein after chondroitinase treatment [38,39]. In wild-type cartilage, low levels of diffuse C0S could be detected in the pericellular and interterritorial matrix. In contrast, bright staining of C0S was observed in cartilage from DNIIR mice. In control cartilage, C4S staining was clearly detected in the pericellular and interterritorial matrix; however, C4S staining was low in DNIIR cartilage, suggesting that reduced sulfation in the matrix resulted in low levels of C4S and correspondingly higher levels of C0S. This staining pattern was previously observed in Papss2^bm/bm^-mutant cartilage [40]. Together these results suggest a defect in sulfation of the cartilage ECM that could result from failure to maintain Papss2 expression in the absence of TGF-β signaling.

## Discussion

Several murine models with altered TGF-β signaling in cartilage show signs of OA, but the biomechanical properties of articular cartilage, which are critical for its load-bearing ability, have not been studied in these mice. Furthermore, the mechanisms by which altered TGF-β signaling results in OA symptoms are not known. Herein we show that articular cartilage from DNIIR mice possess reduced stiffness at 20 weeks of age, as indicated by stress-relaxation indentation tests in comparison to WT mice. To address the function of TGF-β in maintaining the cartilage phenotype, we characterized global gene expression patterns in bovine articular chondrocytes treated with TGF-β. We identified several known TGF-β-responsive genes, including *PRG4*, as well as genes important for posttranslational modifications of the ECM, including *PAPSS2 *and *PLOD2*. Regulation of Papss2 mRNA and protein *in vivo *was confirmed by comparing DNIIR and WT mice. Downregulation of Papss2 in DNIIR mice correlated with reduced critical electrolyte staining for Alcian blue and reduced C4S immunostaining. Nevertheless, immunostaining for the aggrecan core protein was comparable in control and DNIIR mice, suggesting reduced matrix sulfation in the DNIIR cartilage.

The main function of articular cartilage is to act as a cushion in diarthrodial joints by handling various mechanical loads imparted on the joint through normal activity. To characterize the changes in mechanical properties in the DNIIR mutants, we performed a four-step stress relaxation indentation test to measure instantaneous and equilibrium stiffness. Our tests revealed that indentation stiffness is compromised in the DNIIR cartilage, which would affect its load-bearing function. Indentation stress relaxation tests similar to ours have been employed previously to determine mechanical properties of cartilage in mice with osteoarthritic changes [10,22]. Cartilage consists of a solid (matrix), fluid (water) and fixed-charge density, all of which contribute to biomechanical properties of the tissue. Various methods have been used to describe the biomechanical behavior of cartilage, including those that take into account all the constituents of cartilage, in the biphasic [41] and triphasic [42] models of cartilage mechanics. These methods involve rigorous analytical solutions that are beyond the scope of the current study. Although our indentation technique does not delineate the changes in each component of cartilage separately, it still provides us with a structural parameter with which to evaluate the differences in load-bearing ability of articular cartilage.

Papss2 is one of the isoforms of the PAPS synthetase, a bifunctional enzyme responsible for synthesis of PAPS, the universal sulfate donor. Many of the phenotypes observed in humans and mice with mutations in *Papss2 *overlap with those seen in mice with alterations in TGF-β signaling [2,6,20]. Regulation of Papss1 and Papss2 in the liver has been studied [43], but how Papss2, which catalyzes the synthesis of PAPS for sulfation of glycosaminoglycans, is regulated in cartilage is not known. Papss2 mRNA expression starts at 11.5 days postcoitum in mouse cartilage as it condenses and continues in the newborn in all the cartilaginous elements [44]. Interestingly, though its expression is significant in condensing and proliferating chondrocytes, Papss2 mRNA is markedly reduced in hypertrophic chondrocytes. In addition, Papss2 mRNA expression overlaps with that of Sox9, one of the master regulators of chondrogenesis. In this study, we have shown that TGF-β treatment significantly increases *PAPSS2 *expression in bovine chondrocyte cultures. Previously, we found that TGF-β regulates *Papss2 *in mouse embryonic sclerotome [36]. Furthermore, we found significant reduction in Papss2 mRNA and protein levels in the articular cartilage of DNIIR mice, indicating an important role for TGF-β in regulating this enzyme *in vivo*.

Because expression of DNIIR resulted in reduced levels of Papss2, we hypothesized that the ECM in DNIIR cartilage would be hyposulfated. We performed Alcian blue staining using the critical electrolyte concentration principle. When we compared the staining between the genotypes, DNIIR cartilage consistently showed fainter staining, especially in the pericellular and superficial zone matrix. Aggrecan core protein immunostaining showed comparable expression, suggesting the alterations in Alcian blue staining were due to alterations in sulfation. To confirm alterations in sulfation more directly, we performed immunostaining for C4S and C0S. These antibodies were used previously to study sulfation of chondroitin in cartilage [40]. C4S staining was reduced and C0S was increased, suggesting that alterations in TGF-β signaling affect sulfation of GAGs in the matrix. Negatively charged sulfate (SO_3_^-^) and carboxyl (COO^-^) groups help to retain water after exudation caused by loading [13]. Changes in the sulfation of proteoglycans would be predicted to affect how water is retained in the cartilage, which could in turn alter mechanical properties. We hypothesize that altered TGF-β signaling and subsequent downregulation of Papss2 and matrix sulfation affect the biomechanical properties of articular cartilage. It should be noted that changes in Papss2 levels occur in DNIIR mice as early as 6 weeks of age, long before changes in histological or mechanical properties are observed. Turnover of the ECM is very slow, so it is reasonable to propose that the effects of downregulating Papss2 would take a long time to manifest as changes in ECM sulfation or changes in mechanical properties. This suggests TGF-β signaling and regulation of Papss2 expression in mouse cartilage are important in postnatal homeostasis of articular cartilage.

Mutations in other genes of the sulfation cascade, such as chondroitin-4-sulfotransferase 1 (*Chst11*) [45], diastropic dysplasia transporter (*Dtdst *or *Slc26a2*) [46,47] and extracellular heparan endosulfatases (*Sulf-1 *and *Sulf-2*) [48], have also been shown to result in various types of chondrodysplasias, some of which are lethal (mutations in *Dtdst*: achondrogenesis type IB [OMIM:600972]; atelosteogenesis type 2 [OMIM:256050]). *Chst11 *was recently identified as a target of bone morphogenetic protein (BMP) signaling and also was found to contain *cis*-regulatory modules that potentially could respond to TGF-β. We did not identify changes in expression of *CHST11 *or any other sulfotransferase in bovine articular chondrocytes in response to TGF-β in our microarray experiments, although several are spotted on the array and are annotated in the bovine genome. There are some overlapping embryonic phenotypes in the skeletons of mice with conditional deletion of *Tgfbr2 *[2,20] and mutation in *Chst11*, including misshapen vertebrae and failure of the joints to form in the digits; however, the phenotype of the growth plate in *Chst11 *mutants was distinct from that seen in mice with loss of TGF-β signaling and may be due to secondary effects on patterning of both BMP and TGF-β signals. Postnatal maintenance of the articular cartilage was not characterized in mice with a mutation in *Chst11*.

Plod2, a lysyl hydroxylase important for cross-linking collagen, was also regulated by TGF-β. Mutations in *PLOD2 *have been associated with the clinical condition Bruck syndrome 2 [OMIM:609220], which is characterized by abnormal collagen cross-linking in bone, osteopenia and multiple fractures in mice. Unlike mutations in *PAPSS2*, mutations in *PLOD2 *appear to affect bone structure more than cartilage [49,50], although a role in TGF-β-mediated chondroprotection is still possible. Whether alterations in *Plod2 *expression translate to defective cross-linking of type II collagen and alterations in mechanical properties in DNIIR mice was not addressed in this study; however, Sirius Red staining and polarized light microscopy indicated disorganization of collagen fibers in the articular cartilage of DNIIR mice compared to WT controls.

Prg4, a secreted glycoprotein, which is already known to be positively regulated by TGF-β in superficial zone chondrocytes [32] and has a protective effect on cartilage surfaces, was downregulated in the DNIIR mutants at the mRNA level. *Prg4*-knockout mice develop joint disease with age-associated increased joint friction and reduced mechanical properties of the cartilage [51,52]. Decreased Prg4 expression in the DNIIR mutants may also contribute to the reduction in mechanical properties observed.

## Conclusions

In conclusion, TGF-β signaling is required for the biomechanical integrity of articular cartilage. TGF-β upregulates the expression of several matrix-modifying enzymes, including Papss2, which mediates the rate-limiting step of protein sulfation. Tgfbr2 is required to maintain Papss2 mRNA and protein expression *in vivo *as well as sulfation levels in the cartilage ECM.

## Abbreviations

BSA: bovine serum albumin; C0S: chondroitin-0-sulfate; C4S: chondroitin-4-sulfate; DMEM: Dulbecco's modified Eagle's medium; DNIIR: dominant-negative transforming growth factor β type II receptor; ECM: extracellular matrix; Gapdh: glyceraldehyde 3-phosphate dehydrogenase; H & E: hematoxylin and eosin; Hprt: hypoxanthine-guanine phosphoribosyltransferase; LTBP3: latent transforming growth factor β-binding protein 3; Papss2: phosphoadenosine phosphosulfate synthetase 2; PBS: phosphate-buffered saline; PCR: polymerase chain reaction; Plod2: procollagen lysine 2-oxoglutarate 5-dioxygenase 2; Prg4: proteoglycan 4; RT: reverse transcriptase; TGF-β: transforming growth factor β; Tgfbr2: transforming growth factor β type II receptor.

## Competing interests

The authors declare that they have no competing interests.

## Authors' contributions

GR carried out the indentation studies, confirmation of the microarray results and characterization of Papss2 expression and sulfation in wild-type and mutant mice. He also participated in planning the experiments, interpreting the results and writing the manuscript. PS set up the bovine cartilage micromass cultures and carried out the microarray studies. AE helped with the design and interpretation of the indentation studies. RS conceived the study and participated in its design and coordination. She also participated in interpretation of the data and the final writing of the manuscript. All authors read and approved the final manuscript.

## Supplementary Material

Additional file 1 **Genes regulated by TGF-β (*P *< 0.05), up- or downregulated**. Excel file. Click here for file
